# EZH2 inhibition promotes methyl jasmonate-induced apoptosis of human colorectal cancer through the Wnt/β-catenin pathway

**DOI:** 10.3892/ol.2021.12832

**Published:** 2021-06-02

**Authors:** Yao Wang, Li Fan, Chunguo Cui, Yongkun Wang, Tingting Liang

Oncol Lett 16: 1231-1236, 2018; DOI: 10.3892/ol.2018.8779

Subsequently to the publication of the above paper, the authors have requested that the paper be retracted on account of a lack of coordination among different members of the research group who were performing experiments simultaneously, and the fact that an inappropriate cell line was selected for some of the experiments (T24, which is a bladder cancer cell line, was used instead of the SW480 cell line). Consequently, the authors no longer feel confident about the reported results, and consider the conclusions of this study to be unreliable.

The Editor has granted the authors’ request that the paper be retracted, and all the named authors agree to this retraction. The authors sincerely apologize for their errors, and regret any inconvenience caused.

